# Integrative Genomic Data Mining for Discovery of Potential Blood-Borne Biomarkers for Early Diagnosis of Cancer

**DOI:** 10.1371/journal.pone.0003661

**Published:** 2008-11-06

**Authors:** Yongliang Yang, Lakshmanan K. Iyer, S. James Adelstein, Amin I. Kassis

**Affiliations:** 1 Department of Radiology, Harvard Medical School, Harvard University, Boston, Massachusetts, United States of America; 2 Center for Neuroscience Research, Tufts University School of Medicine, Boston, Massachusetts, United States of America; Harvard School of Public Health, United States of America

## Abstract

**Background:**

With the arrival of the postgenomic era, there is increasing interest in the discovery of biomarkers for the accurate diagnosis, prognosis, and early detection of cancer. Blood-borne cancer markers are favored by clinicians, because blood samples can be obtained and analyzed with relative ease. We have used a combined mining strategy based on an integrated cancer microarray platform, Oncomine, and the biomarker module of the Ingenuity Pathways Analysis (IPA) program to identify potential blood-based markers for six common human cancer types.

**Methodology/Principal Findings:**

In the Oncomine platform, the genes overexpressed in cancer tissues relative to their corresponding normal tissues were filtered by Gene Ontology keywords, with the extracellular environment stipulated and a corrected *Q* value (false discovery rate) cut-off implemented. The identified genes were imported to the IPA biomarker module to separate out those genes encoding putative secreted or cell-surface proteins as blood-borne (blood/serum/plasma) cancer markers. The filtered potential indicators were ranked and prioritized according to normalized absolute Student *t* values. The retrieval of numerous marker genes that are already clinically useful or under active investigation confirmed the effectiveness of our mining strategy. To identify the biomarkers that are unique for each cancer type, the upregulated marker genes that are in common between each two tumor types across the six human tumors were also analyzed by the IPA biomarker comparison function.

**Conclusion/Significance:**

The upregulated marker genes shared among the six cancer types may serve as a molecular tool to complement histopathologic examination, and the combination of the commonly upregulated and unique biomarkers may serve as differentiating markers for a specific cancer. This approach will be increasingly useful to discover diagnostic signatures as the mass of microarray data continues to grow in the ‘omics’ era.

## Introduction

Currently, there is a continued need for the discovery of specific blood biomarkers to aid in the noninvasive detection of cancer and the monitoring of the effectiveness of cancer therapy [1]–[3]. Biomarkers are molecules that are indicators of physiologic state and hallmarks of change in a tissue or a bodily fluid during a disease process [3]. Cancer biomarkers in blood are produced by tumor cells and secreted or released into the bloodstream of patients [2]. The measurement of biomarkers in blood is a noninvasive procedure and relatively simple to perform without requirements for special instruments and personnel.

In pace with the post-genomic era, advanced technologies including genomic analysis and proteomics have facilitated the discovery of effective cancer biomarkers [4]–[7]. One advantage of high throughput microarray-based genomic analyses is the capacity to identify a group or cluster of genes overexpressed in tissue or body fluids that encode putative secreted or cell-surface proteins [5], [6], [8]. However, the mining process in microarray-based analysis typically requires in-depth statistical and analytical skills and poses a challenge to researchers who do not possess the required expertise [9]. This paper proposes and presents a biologist friendly and effective microarray-based mining method that facilitates such biomarker discovery.

Recently, we described a rapid, systematic mining strategy to identify overexpressed genes encoding putative hydrolases suitable for our in-house Enzyme-Mediated Cancer Imaging and Therapy (EMCIT) technology, an approach that aims to hydrolyze and precipitate water-soluble, radioactive prodrugs within the extracellular space of solid human tumors for noninvasive diagnosis or therapy [10]–[12]. Herein, we apply a mining strategy that enables the uncovering of potential blood-borne cancer markers in humans based on the combination of an integrated cancer microarray platform, Oncomine [13], and the novel biomarker filtering capability of the Ingenuity Pathways Analysis (IPA) 5.0 program [14]. To identify genes encoding putative secreted or cell-surface proteins in human blood/serum/plasma as potential cancer markers, all genes overexpressed in the extracellular environment of cancerous cells relative to that of corresponding normal cells were filtered and retrieved from the Oncomine database and then imported to and analyzed by the biomarker module of the IPA analysis program. The application of this mining method has led to the identification of hundreds of biomarkers in human tumors: prostate (224), breast (176), lung (244), colon (57), ovary (292), and pancreas (147). The approach also enabled the ranking and prioritization of the identified potential marker genes for overexpression according to normalized absolute Student *t* values.

It has been observed that the expression of common tumor markers related to universal oncogenic processes is stable and unlikely to be affected by the natural progression of cancer [15]. Therefore, the identification of common tumor markers ubiquitously expressed by a few cancer types could increase the sensitivity and specificity of conventional histopathologic evaluation and could serve the general practice of segregating malignant from benign conditions independently of individual taxonomies [16]. Consequently, we determined the biomarkers in common between each two cancer types. The comparison analysis across six different human tumors has led to the detection of 20 to 134 biomarkers as common hits between every two cancer types, suggesting the interrelation of multiple oncogenic pathways. These identified markers may be used as broad molecular pathology tools after validation analysis. Finally, given the common biomarkers, we were able to identify between 3 and 59 potentially unique biomarkers per cancer type. This is unprecedented since one of the key drawbacks to current biomarkers is that most of them are not specific for one cancer type, which can easily lead to false positives in the early detection of cancer. For instance, serum prostate-specific antigen (PSA) level for the screening of prostate cancer was also found to be elevated in patients with breast or lung tumors, leading to 70% failure of early prostate cancer detection [17], [18]. The limited specificity and sensitivity of current early diagnostic biomarkers has greatly restricted their reliability. Therefore, our mining method could serve as a general strategy for discovering more effective individual or grouped specific markers for cancer, hopefully achieving the clinical objective of screening for early and specific detection. To our knowledge, this is the first study examining with an *in silico* genomic approach upregulated marker genes unique for one cancer type.

## Materials and Methods

The data mining strategy for the discovery of cancer biomarkers is based on our recently published methodology exploring the cancer microarray platform, Oncomine, and employing the advanced knowledge bases of Ingenuity Systems, Ingenuity Pathways Analysis, to identify extracellular hydrolases in various types of cancer (unpublished results). Oncomine [13] was chosen because it is a public cancer microarray platform incorporating 264 independent microarray datasets, totaling more than 18,000 microarray experiments, which span 35 cancer types. It unifies a large compendium of other published cancer microarray data, including Gene Expression Omnibus (GEO) [19] and Stanford Microarray Database (SMD) [20], and uniquely provides differential expression analyses comparing most major types of cancer with their respective normal tissues. For example, to identify potentially important genes in a particular cancer, users can perform a “cancer vs. normal” analysis for a given cancer type and those genes that are upregulated in cancer relative to its normal tissue can be retrieved as a list. Each overexpressed gene in the list can then be assessed by the Student *t* test to calculate the *P* or *Q* values (false discovery rate) [21]–[23], mean expression values (mean 1, mean 2), and the normalized Student *t* value. In addition, Oncomine is integrated with the Gene Ontology (GO) annotations filter, which allows users to identify genes with certain biological processes, molecular functions, or cellular locations.

Each of six human tumor types (prostate, breast, lung, colon, ovary, and pancreas) was used in the 〈〈profile search〉〉 function in the Oncomine database to find the available microarray datasets related to the specific cancer type. The analysis type 〈〈cancer vs. normal〉〉 was then applied to filter those microarray datasets exploring cancer relative to its normal tissue. Next, Gene Ontology (GO) annotation keywords implicating the extracellular environment were used to remove those genes unregulated in cancer. Specifically, upregulated genes associated with the following GO terms were searched: 〈〈extracellular space〉〉, 〈〈extracellular region〉〉, 〈〈cell surface〉〉, 〈〈plasma membrane〉〉, and 〈〈integral to membrane〉〉. Each GO annotation term was conceived and consulted in the GO database [24] to deliver the largest number of relevant hits which are likely to encode secreted or cell-surface proteins. Then, a corrected false discovery rate *Q*-value threshold (*Q*≤0.05) was used to filter and retrieve those extracellularly-overexpressed genes with a high confidence of upregulation. Upregulated genes with a *Q* value less than 0.05 were only kept in the list for further analysis and filtering (including the redundant which was removed in the later filtering step).

Human Genome Organization (HUGO) gene identifiers were then used to export the gene lists, in the Microsoft Excel format, into the Ingenuity Pathways Analysis (IPA) program [14], an application that has been built on a large knowledge database acquired by manual curation of full texts of peer-reviewed scientific publications covering information on more than 500,000 mammalian genes or proteins, molecular concepts, and millions of their pathway interactions. IPA biomarker is a module within the new Ingenuity Pathways Analysis 5.0 program which allows the (i) identification and prioritization of the most promising and relevant biomarker candidates according to characteristics that make a gene product a biologically plausible biomarker (a gene or its encoding product has to be linked closely to the pathology of the disease or is on a pathway that is closely linked to the effect of a treatment) (ii) determination of whether a particular gene or protein is detectable in body fluids, and (iii) assessment of whether the candidate biomarker has a strong association with disease processes such as cancer. The retrieved overexpressed genes were imported to the IPA biomarker module, the redundant was resolved, and those genes encoding plausible markers associated with cancer were identified. These biomarkers were further filtered in the IPA biomarker filter module based on the following criteria: fluid – 〈〈blood〉〉 or 〈〈plasma/serum〉〉, disease – 〈〈cancer〉〉, species – 〈〈human〉〉. These filtered blood-based markers were then ranked and prioritized by the abs(*t*) value, where *t* is the normalized Student *t* test value in Oncomine, to reflect the quantitative change of expression level between cancer and its normal tissue, similar to the fold-change value in microarray experiments. The final set of blood-based markers was exported and stored in a Microsoft Excel spreadsheet (see Supplemental file S1) containing the gene product name, synonyms, abs(*t*) value, description, HUGO gene symbol, expression in body fluids, IPA-defined subcellular locations, disease types, and protein family.

Detailed analysis of resulting protein hits was performed retrospectively using iHOP (information hyperlinked over proteins) [11], [25], a program that finds links and cited articles to genes/proteins and identifies the particular gene product if the gene name or synonym name is known. Resulting blood-based markers were checked and consulted by looking up the associated literature references or original publications. Finally, the accuracy of the findings was assessed using control cancer markers either selected as candidate markers by other studies or well known to be clinically useful.

IPA biomarker comparison is another function within the IPA biomarker module, which has the capacity to generate a list of candidate biomarkers common across different diseases [14]. The program can maximally compare the candidate biomarkers for three diseases simultaneously. The filtered biomarkers for each of the six tumor types from the previous step were thus imported and compared between every two tumor types to determine the common biomarkers. The retrieved common biomarkers across the six human tumor types were then used to determine the unique candidate biomarkers per cancer type by the exclusion method.

## Results and Discussion

**Figure 1:**
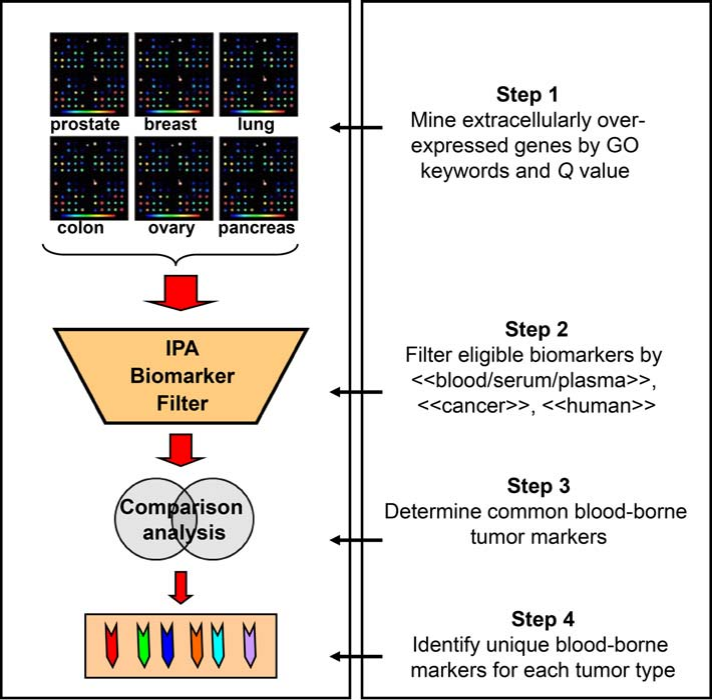
Scheme for mining overexpressed genes in six human tumors to identify potential blood-borne cancer markers. Microarray plates at top represent six tissue types searched in Oncomine platform, including prostate, breast, lung, colon, ovary, and pancreas. Step 1: Overexpressed genes in cancer relative to its corresponding normal tissue were filtered by GO terms and *Q* value cut-off. Step 2: blood borne markers were filtered by 〈〈blood〉〉, 〈〈serum/plasma〉〉, 〈〈cancer〉〉 and 〈〈human〉〉 in biomarker module of IPA program. Trapezoid shape in middle represents biomarker filtering capability of IPA analysis program. Step 3: common markers were determined between every two tumor types. Step 4: unique markers were identified for each cancer type.

The general mining strategy for biomarker discovery reported here is flexible in nature. Researchers may vary the data-filtering criteria according to their own interests. For example, in the first step of the mining process (see Figure 1), they might choose to filter either “upregulated” or “downregulated” genes to identify markers for diagnosis or they choose to filter “differentially expressed” genes in various tumor grades or stages to discover prognostic markers. In the second step, one may choose to filter eligible biomarkers in different biological fluids (such as saliva, tears, and urine) and different species (such as mouse and rat). Moreover, researchers can vary the genomic database and the pathway analysis program. Although our primary interest is to identify markers for human cancer, we believe that this mining strategy can be broadly applied to identify markers for most other types of diseases.

### Identification of eligible cancer markers

**Figure 2:**
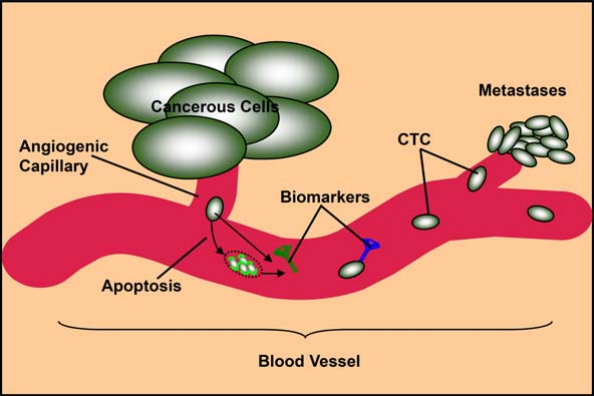
Circulating tumor cells (CTC) and blood-borne markers secreted or released (consequent to tumor-cell fragmentation) in blood vessel.

Data mining of 4 to 15 microarray datasets from the Oncomine platform for genes overexpressed in six human cancers compared with their expression in normal tissues led to the identification of a list of 3,064 to 19,645 upregulated gene expression profiles per cancer type. We were mining for upregulated genes because one of the prevailing hypotheses is that the most promising biomarkers will be overproduced genes or their protein products [26], [27] (this may not be generally true, and other researchers could choose to mine downregulated genes for their specific purpose). Ideally, blood-borne tumor markers would be secreted or otherwise shed into the circulatory system during tumorigenesis. They could be secreted by tumor cells or released consequent to tumor-cell fragmentation (Figure 2). Therefore, we searched for upregulated genes by a combination of controlled Gene Ontology keywords to implicate the extracellular environment (see *Materials and Methods*) in cancerous cells including those encoded proteins bound to or integrated in cell membranes but whose extracellular domains can be found through shedding in the circulation. When the retrieved genes were further filtered by the corrected false discovery rate *Q* (*Q*≤0.05), between 211 and 2,782 genes per cancer type were overexpressed in the extracellular environment of cancerous cells (including the redundant). We used a stringent corrected false discovery rate cut-off value to select significantly upregulated genes and to avoid false predictions arising from experimental variation in different studies. These upregulated genes were imported to the IPA biomarker analysis module and between 165 and 961 genes were identified as eligible candidate markers per cancer type (Table S1).

### Identification of blood-borne cancer markers

The eligible markers that we retrieved from IPA biomarker analysis included those markers upregulated in the tissues or biological fluids of patients with cancer. Next, we filtered the blood-borne markers because they have two major advantages over other types of indicators. First, blood cells communicate with the cells and extracellular matrixes in almost all tissues and organs in the body. Thus, the gene expression profiles of blood cells may reflect the presence of disease in the body [2], [3]. Second, blood sample collection is less invasive and safer, allowing for a larger sample size and repeated sampling to monitor disease progression. From the IPA biomarker filter module, between 57 and 292 blood-borne (blood/plasma/serum) markers were identified per human cancer type (see identified genes in Supplemental File S1). By examining IPA and iHOP knowledge bases [11], [25], we determined that the majority of the blood-borne tumor markers are secreted, or glycosylphosphatidylinositol (GPI)-anchored and integral membrane proteins. The detection of their upregulation in patient blood samples can trigger earlier treatment before tumor growth [2]–[4]. Further, these upregulated signatures could be exploited to understand the pathways related to human cancer and unravel the associations between different tumors. Although the functional mechanism driving the various gene expression profiles in the blood of patients with or without cancer is unclear, the potential clinical utility of these genes or their protein products is emerging. As controls, we have listed below a few blood-derived markers, identified by our work, that have also been selected by other studies as candidate tumor markers or are already being used clinically,

#### Erythroblastic leukemia viral oncogene homolog 2 (ErbB2)

ErbB2 is commonly referred to as Her-2/*neu* by clinicians [28]. It is a cell-membrane-surface-bound tyrosine kinase receptor that is normally involved in the signal transduction pathway leading to cell growth and differentiation [29]. In our study, we identified ErbB2 as a universal blood-borne biomarker for five cancers (prostate, breast, lung, ovary, and pancreas). This is consistent with the findings that the amplification of this gene or overexpression of its protein product is associated with cancers including breast, lung, ovarian, and pancreatic [28]–[31]. In particular, amplification of *ErbB2* gene has been found in 25% to 30% of breast cancer, and it has been formally approved by the FDA as a serum biomarker for the diagnosis of breast cancer [30]. To our knowledge, the overexpression of *ErbB2* gene has not been reported for prostate cancer.

#### Breast cancer 1/2, early onset (BRCA1/BRCA2)

*BRCA1* and *BRCA2* are genes directly involved in cell growth, division, and repair of damaged DNA. The variations in either gene or their protein products have been implicated in prostate, breast, and ovarian cancers [32]. There is also strong evidence to suggest that both genes could be used as predictive markers for the treatment of breast and ovarian cancer [32]–[35]. We found both genes as potential markers in these three tumor types as well as lung cancer. The overexpression of both genes in four human cancers may suggest that they are involved in a generalized phenomenon or functional mechanism in patients with these cancers.

#### Prostate-specific antigen (PSA)

PSA, also known as kallikrein III (KLK3), is a protein produced by the cells of the prostate gland and that is often elevated in the presence of prostate cancer or other prostate disorders [17], [18]. It is a well-known serum biomarker and measurement of serum PSA level is the most effective test currently available for the early detection of prostate cancer [36]. Consistent with other experimental findings [37], we identified PSA as a serum marker for a few tumor types including prostate, breast, and lung cancer, indicating that PSA is not prostate-cancer-specific.

#### Hyaluronan-binding protein 2 (HABP2)

HABP2 is a member of the serine protease family that is found in the plasma/serum and demonstrated to play important roles in cancer invasion and metastasis [38]. A real-time reverse transcriptase polymerase chain reaction (RT-PCR) screening study has demonstrated the specific overexpression of the *HABP2* gene in lung adenocarcinoma, among six candidate marker genes for detection of non-small cell lung cancer [39]. We identified two of these six candidate marker genes, *HABP2* and *CP* (ceruloplasmin), as potential serum marker genes for lung cancer, demonstrating the usefulness of our mining strategy in determining novel, potentially useful, clinical blood markers for human cancer.

#### Insulin-like growth factor-II (IGF-II)

IGF-II encodes a member of the insulin family of polypeptide growth factors that are implicated in the pathogenesis of neoplasm in various tissues [40], [41]. Interestingly, our mining approach identified IGF-II as a potential serum marker for breast, lung, and ovarian cancer. It has been identified by a recent protein microarray experiment based on a blood test as one of four serum markers for discriminating between healthy groups and patients with epithelial ovarian cancer (EOC) [42]. In this proteomic study, IGF-II protein level is reduced in patients with EOC compared with healthy controls, whereas in our gene microarray mining it is upregulated in ovarian cancer. These findings indicate that gene microarray study alone may be insufficient and a more rigorous study involving proteomics experiments or antibody microarrays are necessary to validate the candidate markers at the protein expression level. Nevertheless, our study is consistent with other findings that the upregulation of IGF-II level could be used to diagnose breast [43], [44] and lung [45] cancers.

### Identification of common tumor biomarkers

**Figure 3:**
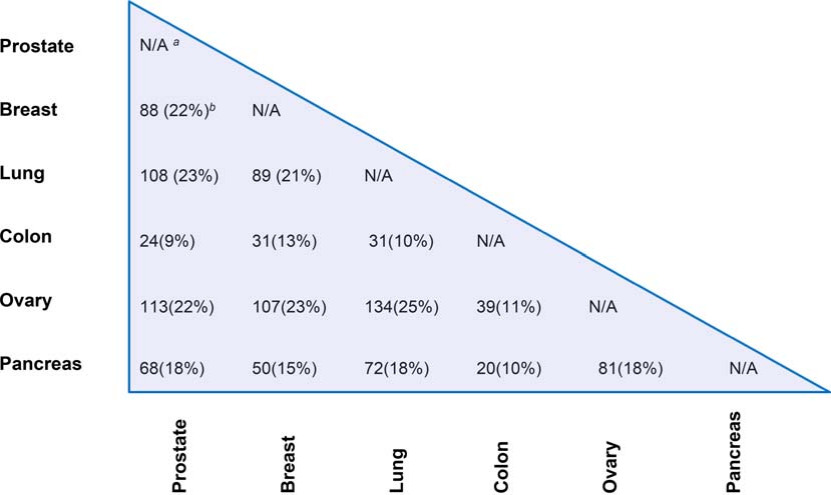
Matrix form for the common markers identified for six human tumors in prostate, breast, lung, colon, ovary and pancreas. *^a^* The comparison of biomarkers between the same tissue type is not available. *^b^* The percent overlap of common markers between every two cancer types is provided in parenthesis.

The tumor markers shared between each two tumor types among the six human tumors were analyzed by the biomarker comparison analysis function of the IPA program and are summarized in a matrix form (Figure 3; see Supplemental File S2). Ovarian cancer has the most markers in common with prostate (113), breast (107), and lung cancer (134) among the 15 different cancer pairs, possibly because we identified ovarian cancer as having the most blood-borne biomarkers (292) (see Table S1) among the six cancer types. Nevertheless, these striking overlaps between different cancer types indicate that the majority of the candidate marker genes may in fact be closely related to multiple oncogenic pathways of cancer metastasis. One of the bottlenecks in discovering appropriate cancer markers is a poor understanding of the pathophysiology of the disease [26], [27], [46]. As such, the universal overexpression of common markers across different human cancers may help in understanding and uncovering the generalized functional mechanisms of tumor growth and invasion. In addition, the commonly upregulated marker genes may assist in relating the relevant markers to the pathogenesis of a particular cancer while any correlation with other cancer types may suggest novel therapeutic targeting strategies. Moreover, common markers might be useful in increasing the sensitivity and specificity of conventional evaluation. For example, the identified universal biomarkers could be used by pathologists for uncovering cancer invasion when comprehensive histologic evaluation is insufficient [15]. To test the hypothesis that common markers shared by various tumor types could be used to distinguish between benign/malignant conditions, we have determined the common set of markers across prostate, breast and lung cancer (see “Supplemental File S3 – prostate, breast, and lung common markers.xls”). Remarkably, after manually consulting the iHOP database and IPA knowledge database, 13 markers out of the common 35 markers (∼1/3) have been literature-confirmed to serve as prognostic markers for the progression and invasiveness of human tumors (Table S2). Although there are no direct evidences that the rest of the 22 common markers can differentiate between benign/malignant conditions, we believe that they may all be involved in cancer metastasis.

### Identification of unique tumor biomarkers

**Figure 4:**
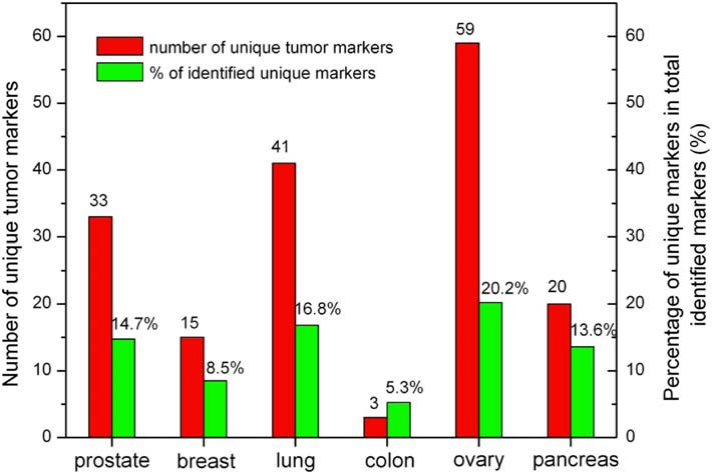
Potential unique markers identified for each human cancer type. Horizonal axis of plot is tumor type, including prostate, breast, lung, colon, ovary, and pancreas cancers. Vertical axis on left is number of unique markers identified for each of six cancer types, represented by red columns in plot. Vertical axis on right is unique marker percentage of total identified blood-borne markers per cancer type, represented by green columns in plot.

In examining the common biomarkers between each two cancer types, we observed 3 to 59 biomarkers exclusive to each cancer type (Table S1, Figure 4; see Supplemental File S2). In effect, less than twenty percent of the total identified blood-based biomarkers per cancer type are unique. A few of the biomarkers reported here have been suggested as putative specific biomarkers by other studies. For example, leptin (LEP), a protein hormone with important effects in regulating body metabolism, has been reported as one of the four specific serum biomarkers for the early detection of ovarian cancer [42]. Our study confirms its potential as a unique blood-borne marker for ovarian cancer. Similarly, we identified matrix metalloproteinase-2 (MMP-2) as a specific biomarker for pancreatic cancer, consistent with the experimental findings showing that its upregulation, compared with that of other metalloproteinases, seems particularly important in the growth and dissemination of pancreatic cancer [47]–[49]. We believe these unique biomarkers could be combined to produce a panel of markers that could improve selectivity and sensitivity for the early diagnosis of cancer.

### Identification of promising top-ranked marker genes

Another application of our mining strategy is the prioritization (according to *t* values) of top-ranked overexpressed marker genes with biological evidence implicating their significant role in cancer. Previously little attention has been paid to their potential as candidate markers or they were missed simply because of the challenge in validating a large pool of candidate genes. These top-ranked marker genes are valuable because they are quantitatively more overexpressed than the other marker genes and thus increase the sensitivity of cancer diagnosis. Those scientists interested in discovering cancer markers could further analyze and validate these candidate markers to make them clinically useful [26], [27]. As examples, we have listed below four top-ranked genes identified in our study.

#### Matrix metalloproteinase-1 (MMP-1) for breast cancer

MMP-1 is a zinc-ion-binding peptidase secreted in the extracellular space and involved in the breakdown of extracellular matrix. Upregulation of MMP-1 mRNA and elevated levels of its protein have been observed in several cancers [50]. However, in the past, most studies have focused on its diagnostic significance for lung cancer [51] or its prognostic significance for colorectal cancer [52]. Notably, our study identified MMP-1 as the most upregulated marker gene for breast cancer, opening up the possibility, after follow-up validation studies, for its use as a putative predictive marker in screening for breast cancer.

#### CD44 for colorectal cancer

*CD44* encodes a cell-surface glycoprotein involved in cell–cell interactions, cell adhesion, and migration. This protein participates in a wide variety of cellular functions, including lymphocyte activation and tumor metastasis [53]. In the IPA knowledge bases and iHOP database, there is evidence implicating the expression of this protein in colorectal cancer [53], [54]. We identified *CD44* as the most upregulated marker gene for colon cancer among 57 putative biomarkers. Thus, CD44 could be another promising diagnostic marker for screening colorectal cancer.

#### Ceruloplasmin (CP) for ovarian cancer

*CP* encodes an extracellular metalloprotein that binds most of the copper in plasma and regulates cellular iron-ion homeostasis in the circulation [55]. In the past, little attention was paid to its role in human neoplasia, although it had been suspected that the expression of ceruloplasmin protein is related to ovarian cancer [56]. We identified *CP* as the second most upregulated gene for ovarian cancer, indicating its potential as a promising serum marker.

#### Notch homolog 4 (NOTCH4) for pancreatic cancer

*NOTCH4* encodes a member of the Notch protein family that is involved in the Notch signaling network and presented on the cell surface as a heterodimer. This protein functions as a receptor for membrane-bound ligands and may play a role in vascular, renal, and hepatic development [57]. Notch pathway components and Notch target genes are upregulated in invasive pancreatic cancer cells [58]. A more detailed gene expression profiling study has demonstrated that the mRNA of *NOTCH4* is highly upregulated in pancreatic adenocarcinomas [59]. We identified *NOTCH4* as the most specific upregulated marker gene for pancreatic cancer, strongly suggesting its potential for the diagnosis of invasive pancreatic cancer.

### Conclusion

We present and apply an integrative mining strategy to identify overexpressed genes which encode secreted proteins as putative blood-borne biomarkers for six common human tumors. The mining strategy is based on the combination of a public cancer microarray platform, Oncomine, and the novel biomarker filtering capabilities of the IPA pathways analysis program. Our mining strategy is uniquely biologist friendly and flexible so that it can be broadly applied to the discovery of biomarkers for many other disease types. The detection of numerous cancer marker genes that are clinically useful or experimentally identified supports the effectiveness of our strategy. We have determined the shared markers between every two tumor types across the six selected human tumors; these commonly upregulated marker genes may serve as a molecular tool to complement conventional blood-assay examination and distinguish between benign/malignant conditions. The finding that the majority of the identified marker genes for one cancer type are shared by the other cancer types suggests the complexity of human cancer and the close relationship of multiple oncogenic pathways. Finally, we have identified unique biomarkers for each cancer type. We propose that in combination they might serve as diffentiating markers for a specific cancer. We have attempted to identify rapidly by an *in-silico* approach significantly upregulated genes as potential blood-borne markers for human cancers. We hope this study will stimulate further experimental studies to define clinically useful diagnostic or prognostic fingerprints in human blood [60]. Nonetheless, this approach will be increasingly useful to discover putative diagnostic signatures as the mass of microarray data continues to grow in the ‘omics’ era.

## Supporting Information

Table S1 Number of blood-borne cancer markers identified in six common human tumors.
(0.04 MB DOC)

Table S2 Literature-confirmed marker genes (among 35 common markers across prostate, breast and lung cancer) that are prognostic of benign and malignant disease in patients.
(0.04 MB DOC)

Supplemental File S1 Ranked blood-borne biomarkers for six human tumors
(0.83 MB XLS)

Supplemental File S2 Unique blood-borne biomarkers for six human tumors
(0.16 MB XLS)

Supplemental File S3 Prostate, breast and lung common markers
(0.02 MB XLS)
