# Correction: Integrative Genomic Data Mining for Discovery of Potential Blood-Borne Biomarkers for Early Diagnosis of Cancer

**DOI:** 10.1371/annotation/9fbf88b3-5041-4a5e-b569-bc6c0e5f9c29

**Published:** 2009-01-26

**Authors:** Yongliang Yang, Pavel Pospisil, Lakshmanan K. Iyer, S. James Adelstein, Amin I. Kassis

Dr. Pavel Pospisil should be included in the author byline instead of the Acknowledgments. He should be listed as the second author, and his affiliation is 1: the Department of Radiology, Harvard Medical School, Harvard University, Boston, Massachusetts, United States of America. The contributions of this author are as follows: Conceived and designed the experiments, contributed reagents/materials/analysis tools.

The corrected citation should read: Yang Y, Pospisil P, Iyer LK, Adelstein SJ, Kassis AI (2008) Integrative Genomic Data Mining for Discovery of Potential Blood-Borne Biomarkers for Early Diagnosis of Cancer. PLoS ONE 3(11): e3661. doi:10.1371/journal.pone.0003661

