# Supplementary material for: Integrative Genomic Data Mining for Discovery of Potential Blood-Borne Biomarkers for Early Diagnosis of Cancer
Source: PLoS One. 2008 Nov 6;3(11):e3661. doi: 10.1371/journal.pone.0003661 (PMC2575235; doi:10.1371/journal.pone.0003661)
Supplement: Table S1 — Number of blood-borne cancer markers identified in six common human tumors. (0.04 MB DOC) [file pone.0003661.s001.doc]

**Table S1.** Number of blood-borne cancer markers identified in six common human tumors.*^a^*

| **Tumor type** | **Prostate** | **Breast** | **Lung** | **Colon** | **Ovary** | **Pancreas** |
| --- | --- | --- | --- | --- | --- | --- |
| **Oncomine:** number of microarray datasets | 82 | 170 | 95 | 41 | 77 | 14 |
| Datasets remaining after filtering by analysis type <<cancer vs. normal>> | 13 | 6 | 15 | 4 | 7 | 5 |
| Total number of measured genes *^b^* | 181,361 | 121,400 | 156,767 | 35,195 | 97,581 | 149,309 |
| Total number of upregulated genes (including the redundant) | 13353 | 12297 | 18188 | 3064 | 19645 | 3402 |
| Entities filtered by Gene Ontology ^c^ with *Q* values cut-off of 0.05 *^c^* | 1929 | 1055 | 2233 | 211 | 2782 | 566 |
| **IPA**-biomarker analysis: eligilbe biomarker | 943 | 855 | 880 | 165 | 961 | 481 |
| **IPA**-biomarker filter module: **fluid**: <<plasma>>, <<serum>>, <<blood>>; **disease**: <<cancer>>; **species**: <<human>> | 224 | 176 | 244 | 57 | 292 | 147 |
| Unique biomarkers ^d^ | 33 | 15 | 41 | 3 | 59 | 20 |
| *^a^*Completed by 12/01/07; *^b^* sum of measured genes in all datasets filtered by <<cancer vs. normal>>; *^c^* controlled GO keywords include <<extracellular space>>, <<extracellular region>>, <<cell surface>>, <<plasma membrane>> and  <<integral to membrane>>. ^d^ the unique biomarkers were determined by the “exclusion” method based on the common biomarkers across six different tumor types | | | | | | |
