# Supplementary material for: Integrative Genomic Data Mining for Discovery of Potential Blood-Borne Biomarkers for Early Diagnosis of Cancer
Source: PLoS One. 2008 Nov 6;3(11):e3661. doi: 10.1371/journal.pone.0003661 (PMC2575235; doi:10.1371/journal.pone.0003661)
Supplement: Table S2 — Literature-confirmed marker genes (among 35 common markers across prostate, breast and lung cancer) that are prognostic of benign and malignant disease in patients. (0.04 MB DOC) [file pone.0003661.s002.doc]

**Table S2.** Literature-confirmed marker genes (among 35 common markers across prostate, breast and lung cancer) that are prognostic of benign and malignant disease in patients.

| **Gene/Protein** | **Prognostic values** | **Confirmed by literatures** |
| --- | --- | --- |
| ADAM15 | Prostate cancer metastasis | Najy et al. (2008) *Cancer Res.* 68(4):1092-9. |
| ADAM28 | Lung cancer progression | Takashi et al. (2006) *Int. J. Cancer* 118:263-273. |
| CXCR4 | Lung cancer progression | Su et al. (2005) *Clin. Cancer Res.* 11(23):8273-80. |
| FSTL1 | Prostate cancer progression | Henshall et al. (2003) *Cancer Res.* 63: 4196-4203. |
| GJB2/CX26 | Breast cancer progression | Yasuto et al. (2007) *Breast Cancer Res. Treat* 106:11-17. |
| INHBA | Prostate cancer progression | Sardana et al. (2007) *Clin. Chem.* 53(3): 429-437. |
| ISG15 | Breast cancer progression | Bektas N. et al. (2008) *Breast Cancer Res.* 10(4):R58. |
| CD24 | Breast cancer metastasis | Kristiansen et al. (2003) *Clin. Cancer Res.* (9):s4906-13. |
| MMP10 | Prostate cancer progression | Riddick et al. (2005) *Br J Cancer* 92: 2171-80. |
| MMP13 | Breast cancer invasiveness | Zhang et al. (2008) *BMC Cancer* 8:83. |
| NCAM1/CD56 | Lung cancer metastasis | Yun et al. (2005) *Ai Zheng* 24(9):1140-3. |
| SPINT2/HAI-2 | Breast cancer invasiveness | Parr et al. (2004)  *Clin. Cancer Res.* 10:202-211. |
| TACSTD1/Ep-CAM | Prostate cancer progression | Poczatek et al. (1998) *J. Urol.* 162:1462-1466. |
